# A Global View on Block Copolymers

**DOI:** 10.3390/polym12040869

**Published:** 2020-04-10

**Authors:** Massimo Lazzari, Mercedes Torneiro

**Affiliations:** 1 Departamento de Química Física, Facultade de Química and Centro Singular de Investigación en Química Biolóxica e Materiais Moleculares (CIQUS), Universidade de Santiago de Compostela, 15782 Santiago de Compostela, Spain; 2 Departamento de Química Orgánica, Facultade de Química, Universidade de Santiago de Compostela, 15782 Santiago de Compostela, Spain; mercedes.torneiro@usc.es

**Keywords:** self-assembly, nanomaterials, drug delivery, PS-*b*-PMMA, poly(ethylene oxide)

## Abstract

In this systematic review, a total of 45,143 publications on block copolymers, issued between 1952 and 2019, are analyzed in terms of number, source, language, institution, country, keywords, and block copolymer type, to find out their evolution and predict research trends. The number of publications devoted to block copolymers has been growing for over six decades, maintaining a consistent level throughout the last few years. In their majority, documents came out of the United States, although more recently, Chinese institutions are those displaying the largest production. Keywords analysis indicated that one-third of the publications concerned synthesis, around 20% explored self-assembly and morphological aspects, and another 20% referred to block copolymer applications in solution. In particular, 2019 confirmed the expansion of studies related to drug delivery, and in minor extent, to a deeper view of self-assembling. Styrene–butadiene–styrene block copolymer was the most popular in studies covering both basic and industrially oriented aspects. Other highly investigated copolymers are PEO-*b*–PPO-*b*–PEO (Pluronic©) and amphiphilic block copolymers based on polycaprolactone or poly(lactic acid), which owed their success to their potential as delivery vehicles. Future trending topics will concern nanomedicine challenges and technology-related applications, with a special attention toward the orientation and ordering of mesophase-separated morphologies.

## 1. Introduction

Block copolymers are a class of polymers formed by two or more homopolymer fragments joined together by covalent bonds. Due to their usual reciprocal insolubility, the chemically different blocks easily segregate to form intermolecular phase-separated morphologies either at the solid state or in solution. The size, the periodicity in the nanoscale, the ordering, and the orientation of such microphases in the solid state may, in principle, be controlled by fine-tuning block molecular weights, molecular weight distribution, composition, the interaction parameters between block components, and also the conditions of self-assembly [1]. For block copolymer thin films, additional interactions with the eventual substrate and free interfaces should also be considered [2], whereas for the assembling in solution, the formation of differently shaped micelles, lamellar structures, or vesicles also depends on solubility parameters, the possible crystallinity of the core-forming block, the addition of further molecular components, etc. [3].

All these, nowadays, apparently basic considerations on block copolymers are the result of decades of investigations and publications, which also had the effect to enable their use as a tool for nanomaterial fabrication (after some processing or as templates), model for behavioral studies, vehicle for drug delivery, additive in photovoltaic cells, precursor for nanocarbons, industrial compatibilizing agent, and many other potential applications. For an exhaustive view of such fundamental or applicative topics, the reader will be referred to specialized references throughout this work or may refer to recently published reviews [4,5,6].

This systematic review aims to analyze most of the bibliographic data concerning the worldwide block copolymer research, in terms of number of publications, source, language, institution, country, keywords, and block copolymer type. Using tools and methodologies typically applied in bibliometry, noteworthy conclusions may be drawn, from the identification of the main countries and institutions working in the field to the evaluation of milestones and specific topics and their evolution. That is with the presumption of being able to understand where the research field comes from, how the actual situation is, and predict where it is going. Keeping in mind the title of a famous talk by Feynman [7], the final purpose of the review is to give an answer to the question: “Is there still plenty of room at the bottom” for block copolymers?

## 2. Methods

A complete search from the Elsevier Scopus abstract and citation database was carried out using the search query [KEY(“block copolymer*”) OR TITLE(“block copolymer*”]. On February 26, 2020, the search resulted in 45,481 documents that after limiting the timescale to 2019 were reduced to 45,143. The use of different search parameters would vary the results, and also the choice of another database would result in a different set of documents. Naturally, the query may have some inherent gaps due to the criteria of selection of keywords by the authors or the editorial, and therefore it does not include all the publications concerning the topic of interest. As an example of qualitatively important documents that were left out, it is important to mention pioneering works by Szwarc and co-workers [8,9], due to the use of the term ‘block polymer’ instead of ‘block copolymer’, which was later on adopted by polymer scientists. In addition, datasheets of early documents only show basic information, possibly including the abstract, but without reporting keywords. At the same time, the database suffers from an incomplete registration of books, and, e.g., does not include the fundamental book cited as reference 1. Despite such limitations, we assume that the analyzed set is big enough to supply accurate information on block copolymer investigations, and the lack of some specific documents (even the very important ones) do not statistically affect the overall observations and conclusions.

In this systematic review, elaborated taking into account the PRISMA-P protocol [10], the following features were studied: number of publications, their distribution by document type, language, institution, country, source title, highly cited publications, keywords, and block copolymer type. Only in the case of the selection of relevant block copolymer types, a different search query was used. As an example, a complete search of all the documents concerning the diblock copolymers formed by a polystyrene block and a poly(methyl methacrylate) block (generally named PS-PMMA) was carried out using the query [TITLE-ABS-KEY (“polystyrene-block-poly(methyl methacrylate)” OR “polystyrene-block-polymethylmethacrylate” OR “polystyrene- block-polymethyl methacrylate” OR “PS-b-PMMA” OR “poly(methyl methacrylate)-block-polystyrene” OR “polymethylmethacrylate-block-polystyrene” OR “polystyrene-block-polymethyl methacrylate” OR “PMMA-b-PS”)].

In all the cases, data were downloaded from Scopus as comma-separated values (CSV) files and conveniently processed by Microsoft Excel. Alternatively, any other spreadsheet could be used for clean up and transformation. Data are shown as bar, pie, or line charts, as well as area cartogram, bibliometric networks, or word clouds. In particular, geography data were obtained from the Wikipedia public domain through Microsoft Excel Office 365. Bibliometric networks, namely communities of countries and associations, were visualized using the VOSviewer version 1.6.14 [11] on the basis of co-authorship, which is one of the most tangible and well-documented forms of scientific collaboration. Each node represents a country author and each link represents co-authorships. Node dimensions are directly proportional to the total number of publications per country, whereas clustering fine-tuning criteria are reported in the figure caption. WordArt was used to create word clouds of keywords [12]. The keyword size is directly proportional to the number of documents. 

## 3. Results

### 3.1. Evolution of the Scientific Output and Impact

**Figure 1:**
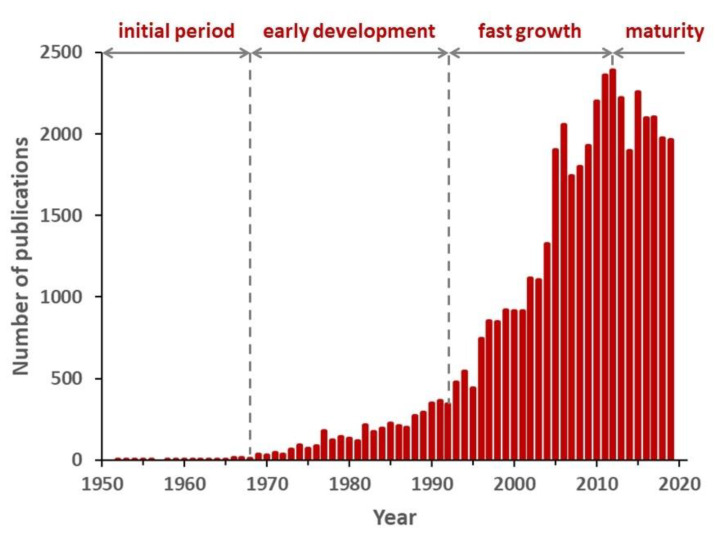
Number of publications between 1950 and 2019.

The evolution of the number of publications on block copolymers from the very beginning, with the first publication directly mentioning block copolymer synthesis in 1952 [13] to 2019 is shown in Figure 1. Four different trends are clearly visible. During the first period, from the beginning to 1968, the publications on this subject were infrequent, reaching a maximum of 13 in 1967. The second, until 1992, corresponded to an increase up to around 350 publications per year, whereas in the third, the interest for block copolymers rose dramatically during 20 years, reaching more than 2300 publications per year by 2012, this being a great indicator of the importance of this field in current polymer science research. Finally, from 2012 onwards, the scientific output was fluctuating around a little lower number, possibly suggesting the achievement of a maturity level. The four periods account for 0.1%, 8.8%, 58.9%, and 32.2% of a total of 45,143 documents, respectively. 

**Figure 2:**
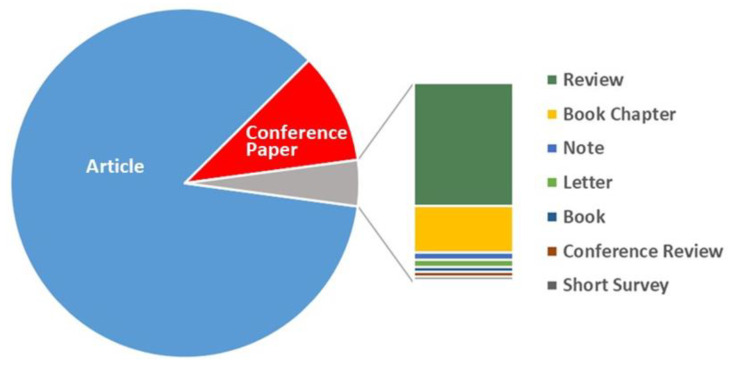
Distribution of document types for block copolymers.

Most publications on block copolymers are articles (85.3%) or conference papers (10.3%) (Figure 2). In third position are reviews, which are usually understood to be manuscripts that resume previous publications in a field without using original material; these represent a huge 2.6% of publications. It is worth mentioning that the first document of this type, published in 1971 [14], cited 235 works, nicely focusing on the last developments on synthesis, characterization, and properties, also including an early view on commercial uses of block copolymers. As a comparison, the year with the highest number of reviews was 2016, with 87. Other studies appear at a much lower frequency, such as book chapters (1.0%) or books (0.1%). Furthermore, as most of the studies were published in international journals, mostly English speaking, English is the predominant language; it was found in 93.9% of documents (results not reported in the form of a table or figure). Among the minority languages, the use of Chinese and Japanese, with 2.2% and 2.0%, respectively, envisages a strong presence of these countries.

In order to discuss the impact of publications, their citations are briefly analyzed. To date (by February 17, 2020), the documents dealing with block copolymers have an h-index of 343, with 41 articles or reviews having more than 1000 citations. The three most influent publications have ca. 10,000 [15], ca. 3300 [16], and ca. 3100 [17] citations, respectively. On the other side, the top three books [18,19,20] accumulate a much lower number of citations, ca. 750, 300, and 250 (including 45 citations of individual chapters), respectively. Similarly to other research fields, original articles and periodic reviews have a high impact on the scientific community, whereas the importance of handbooks or textbooks, usually conceived to spread basic knowledge or to give practical indications providing ready references, does not seem to be as easy to be measured by this citation parameter.

### 3.2. Geographical Distribution of Publications

**Figure 3:**
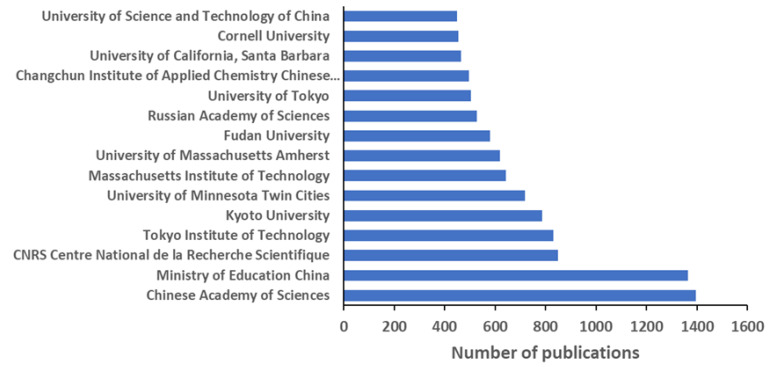
Top 15 institutions by number of scientific publications on block copolymers. The most cited publication of each institution is reported as reference by the decreasing order of number of publications [21,22,23,24,25], [17], [26,27,28,29,30,31], [15], [32], [33].

All the top 15 institutions (Figure 3), each accounting for more than 400 publications, are nonprofit organizations, either universities or large public institutions dedicated to multidisciplinary research. Only two are Europeans—the French Centre National de la Reserche Scientific and the Russian Academy of Science—while five are from the United States of America, five are Chinese, and three are Japanese. The Tokyo Institute of Technology was the most productive university [21], after three large fundamental science national agencies. Among the 158 institutions with more than 120 publications until 2019, 33 (around 20%) are from the United States. In a similar way, the companies dedicating the most efforts to disseminate knowledge on block copolymers were the American IBM (Almaden Research Center), The Dow Chemical Company, and Exxon Mobil Corporation, with a lower number of documents by the French Arkema Group and the German BASF SE. 

**Figure 4:**
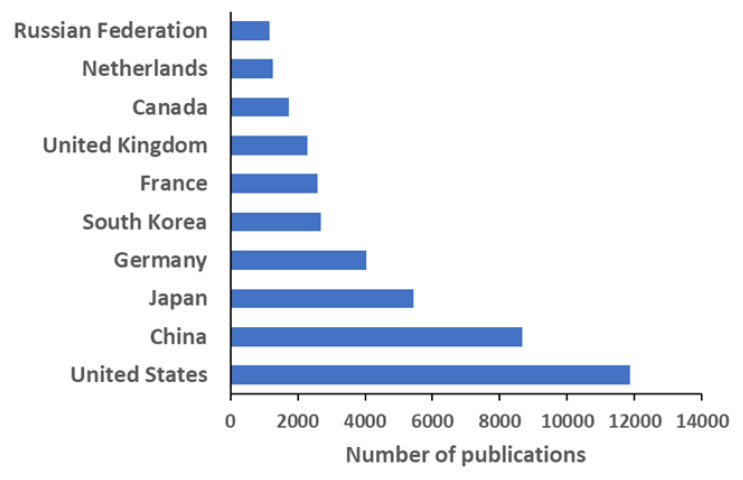
Top 10 countries by number of scientific publications on block copolymers.

**Figure 5:**
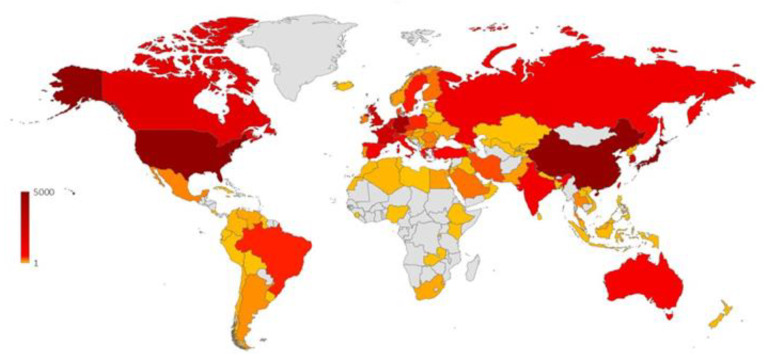
World map with main countries and their number of publications on block copolymers. The deep red color indicates the countries with the biggest number of publications, the yellow indicates smaller numbers of publications, and the gray indicates the countries without any publications.

**Figure 6:**
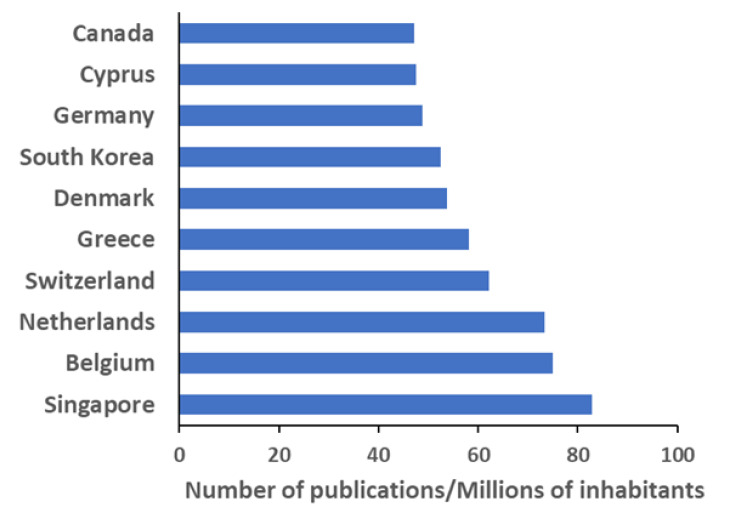
Top 10 countries in normalized scientific production, as number of publications per million of inhabitants.

The apparent leading position of the United States’ investigations is confirmed by the analysis by country (Figure 4), where this entry represents around 26% of the publications. China and Japan are in the second and third positions, respectively. Other countries as Germany, South Korea, France, and the United Kingdom are well positioned in the top 10 list, and they account for more than 2000 publications each; except in the case of France, their institutions are not included amongst the top 15. This situation is similar to that of the United States, where the absence of a strong national agency formed by several institutes working on the same subject is supplied by a widespread interest in many institutions with a proportionally smaller individual output. In Figure 5, the scientific production of each country is color highlighted in a world map. Deep red indicates countries with more than 4000 publications, pale reds refer to countries with a smaller number of documents (in the range of 300–3000), down to oranges and light yellow, the latest indicating countries with at least 1 document. This field of study appears as especially relevant in North America, Europe, and the Far East, with some peaks of scientific production also in Turkey, Australia, Brazil, and India. Interestingly, normalizing the number of documents by the country population, the ranking changes completely (Figure 6). The top position goes to Singapore with more than 82 publications per million inhabitants, followed by Belgium and the Netherlands, with more than 70. The leadership of this small country is essentially due to the excellent activity by a single recently formed institution, the National University of Singapore (the most cited publication of those therein produced is listed as reference [34]). On the other side, the following European countries have a consolidated tradition of research on polymers, which is well exemplified by their leading institutions, namely the University of Liège (Belgium) [35], the Eindhoven University of Technology (the Netherlands) [36], the ETH Zurich (Switzerland) [37], the University of Athens (Greece) [38], and the Technical University of Denmark [39], respectively. In addition, in the case of the bigger countries of this series, i.e., South Korea and Germany, their production was ascribed to the sharp activity of recent institutions such as the Pohang University of Science and Technology (South Korea) [40] or the tradition of an established research center such as the Max Planck Institute for Polymer Research (Germany) [41], respectively.

**Figure 7:**
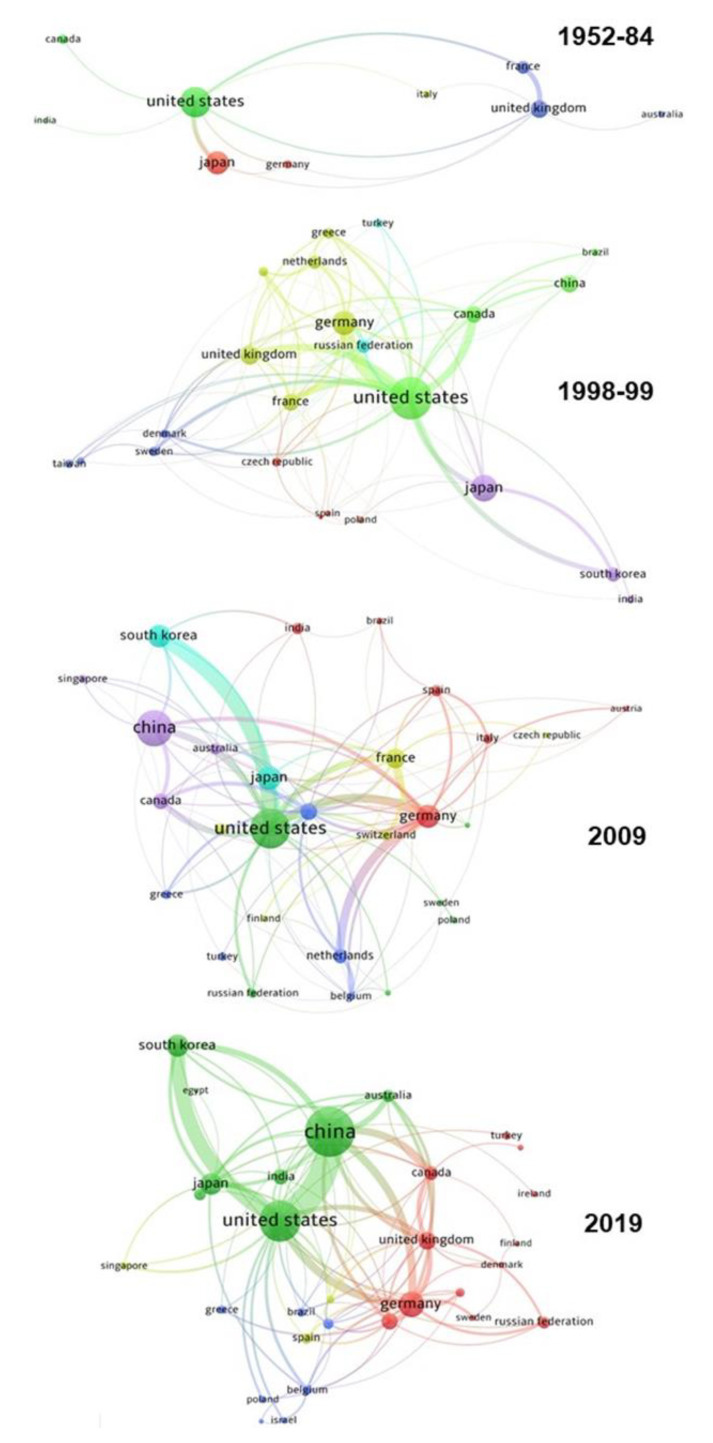
Evolution of the communities of countries and their associations in publications on block copolymers, in the period 1952–1984, 1998–1999, and during the years 2009 and 2019. Only countries with at least 10 documents and 5 overall links are shown, except for the period 1952–1984 where the threshold is just one link.

Figure 7 shows the distribution of communities of countries that published at least 10 documents on block copolymers in four well-differentiated periods, each of them analyzing a comparable number of documents (in the approximate range 1800–1950): between 1952 and 1984, in 1998–1999, 2009, and 2019. Only nine countries appear in the seminal period, with three significant communities centered on the United States, Japan, and the United Kingdom, and a very limited number of links between countries. The strongest connections—between the United States and Japan or between the United Kingdom and France—account for only 9 and 10 documents respectively, over a total of 1795. These results show that early investigations on block copolymers were conceived and developed essentially at national level, often within a single institution, essentially without the need of any collaboration. Moving to 1998–1999, the evolution of the sector toward the development of relationships is clear: 22 countries are distributed in three main communities centered in the Unites States, Japan and Germany, with stronger links (in the range 20–39) between the United States on one side and Germany, the United Kingdom, France and Canada on the other side. In the following distributions, i.e., 2009 and 2019, a much larger number of documents is the outcome of international collaborations. With respect to older distributions, the countries with the biggest potential are in the center of greater communities. The links between Chinese and American institutions grew up to more than 200 in 2019, and China and the United States became the dual center of block copolymer research.

### 3.3. Journals

**Figure 8:**
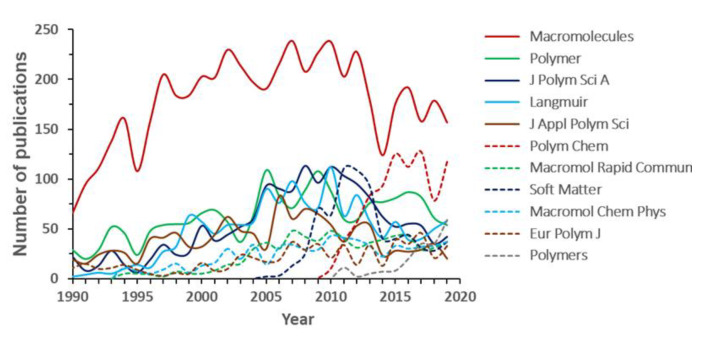
Trend for the main journals (publications between 1990 and 2019).

The evolution of the 10 journals that published the highest number of articles on block copolymers over the last 30 years, and those standing in the top 5 positions in 2019 is visible in Figure 8. All over the analyzed period, the first place was occupied by *Macromolecules* [27,29,35,41], whose trend roughly corresponds to the evolution of the total number of publications visible in Figure 1 (see e.g., the correspondence between the relative minima in 1995 and 2014). This journal alone accounted for 17% of publications in 1990, and its relevance in the field grew up to 29.6% in 1994. Such level was maintained for a few years, then decreasing slowly to less than 10% from 2010 on. The initial faster than average increase of interest may be explained through the ability of the (editorial office of the) journal to intercept from the beginning the potential of block copolymers. In the following years, the transition to applicative topics and the availability of basic and characterization tools to a wider research community made other journals more competitive to collect a larger output. As an example, journals with a more general scope widely focused on materials, as *Langmuir* or *Soft Matter*, conveyed the interest in block copolymers toward a better comprehension of interface phenomena [42] and materials design and fabrication [43], respectively. Other general polymer science journals as *Polymer*, *Journal of Polymer Science Part A: Polymer Chemistry*, *Macromolecular Chemistry and Physics*, *Macromolecular Rapid Communications* and *European Polymer Journal* showed trends similar to *Macromolecules*, although with less emphasized changes. During the last years, top positions were also occupied by recently established journals, such as *Polymer Chemistry* and the open access journal *Polymers*. 

Amongst the most influential journals that published a quantitatively small but qualitatively important series of articles, it is worth citing the multidisciplinary journals *Nature* (21 documents) [13,40,44] and *Science* (43) [15,26], the chemistry journals *Journal of the American Chemical Society* (463) [45] and *Angewandte Chemie* (357) [32,46], and the materials science journals *Nature Materials* (40) [24,36,47] and *Progress in Polymer Science* (109) [14,48].

### 3.4. Keywords and Block Copolymer Types

**Figure 9:**
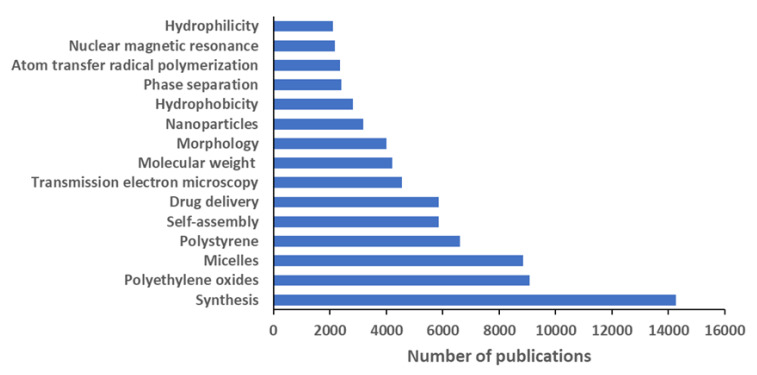
Top 15 keywords in scientific research on block copolymers. ‘Synthesis’ refers to the sum of the keywords ‘copolymerization’, ‘polymerization’, ‘synthesis’, and ‘synthesis (chemical)’; ‘polyethylene oxides’ also refers to the sister keyword ‘polyethylene glycols’; ‘micelles’ also refers to the keyword ‘micelle’; ‘polystyrene’ also refers to ‘polystyrenes’; ‘self-assembly’ also refers to ‘self assembly’; ‘drug delivery’ includes the keywords ‘drug delivery system’, ‘drug delivery systems’, ‘controlled drug delivery’, ‘drug carrier’, and ‘drug carriers’; ‘transmission electron microscopy’ also refers to ‘high-resolution transmission electron microscopy’.

The analysis of keywords was carried out by discarding all those that were obvious, such as ‘block copolymer’, ‘block copolymers’, ‘copolymer’, ‘polymer’, or ‘article’, and gathering together those that are very similar to each other in a single unifying term. As an example, the keyword ‘synthesis’ also includes ‘copolymerization’, ‘polymerization’, and ‘synthesis (chemical)’. Figure 9 shows the top 15 keywords that appeared in more than 2000 block copolymer-related documents. These terms are tentatively gathered in 7 groups. The most ubiquitous keyword group refers to block copolymer preparation and includes ‘synthesis’ and ‘atom transfer radical polymerization’, for a total of around 33% of publications. A second group regards molecular and structural characterization techniques (20.3%), including the terms ‘molecular weight’, ‘transmission electron microscopy’, and ‘nuclear magnetic resonance’, and it is related to basic investigations as well. A third group includes the keywords ‘self-assembly’, ‘phase separation’, and ‘morphology’, which may be found in either basic or applicative publications (20.5%). Another group includes two of the most important block copolymer properties, such as ‘hydrophobicity’ and ‘hydrophilicity’ that were investigated in around 9% of publications.

The keyword ‘nanoparticles’ concerned both the direct use of block copolymers for controlling metal nanoparticle fabrication and especially their use for the formation of polymeric nanoparticles in solution, which are intended as micelles, nanospheres, nanocapsules, and polymersomes. Therefore, ‘nanoparticles’ and ‘micelles’ were considered together and jointly account for 20.4% of publications, whereas the use of block copolymers for ‘drug delivery’ is by far the most investigated application (around 13%). In addition, one of the keywords of the last group formed by the two most common class of copolymers as keywords, namely ‘polyethylene oxides’ (20.0%), mostly refers to their application in solutions. Finally, the ‘polystyrene’-based copolymers include investigations on self-assembly and applications either in solid state or in solution and appear in almost 14% of documents. 

**Figure 10:**
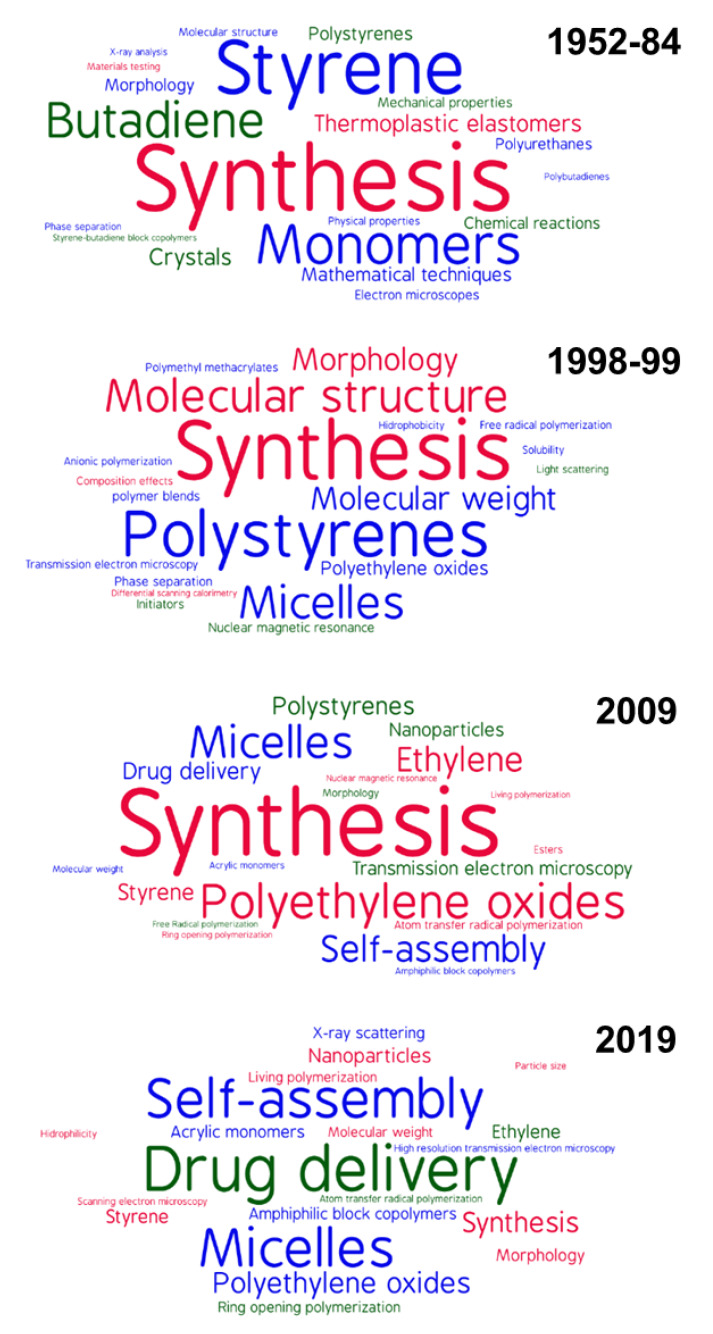
Word clouds of author keywords and their associations in publications on block copolymers, in 1952–1984, 1998–1999, 2009, and 2019. Every word cloud considers the top 20 keywords of the period, with sizes directly proportional to the number of publications.

A first qualitative attempt to follow the change in the focus of investigations over almost 70 years is visualized by the word clouds of the top 20 keywords in the periods 1952–1984, 1998–1999, 2009, and 2019 (Figure 10), each of them obtained analyzing a comparable number of documents (in the approximate range 1800–1950). During the first period, the interest focused on essential aspects as those related to synthesis and characterization, being ‘styrene’, ‘butadiene’, and related keywords (namely ‘polystyrenes’, ‘polybutadienes’, ‘thermoplastic elastomers’, and ‘butadiene–styrene block copolymers’), which is a clear reference to the few available block copolymers. In 1998–1999 and later in 2009, some other polymers (‘polymethyl methacrylates’ and especially ‘polyethylene oxides’), polymerization methods (‘anionic polymerization’ and ‘atom transfer radical polymerization’), and improved characterization techniques (e.g., ‘light scattering’ and ‘transmission electron microscopy’) appeared as top keywords or showed an increase in their frequency of use. The word cloud of 2009 also shows the growing concerns about the behavior of block copolymers in solution, through the size of the keywords ‘micelles’, ‘nanoparticles’, ‘drug delivery’ and ‘amphiphilic block copolymers’. 2019 confirmed such evolution and ‘drug delivery’ stands as the biggest word, surrounded by strictly related keywords as comparably sized ‘micelles’ and ‘self-assembly’ and smaller ‘polyethylene oxides’ and ‘nanoparticles’, possibly marking the route for the following years.

**Figure 11:**
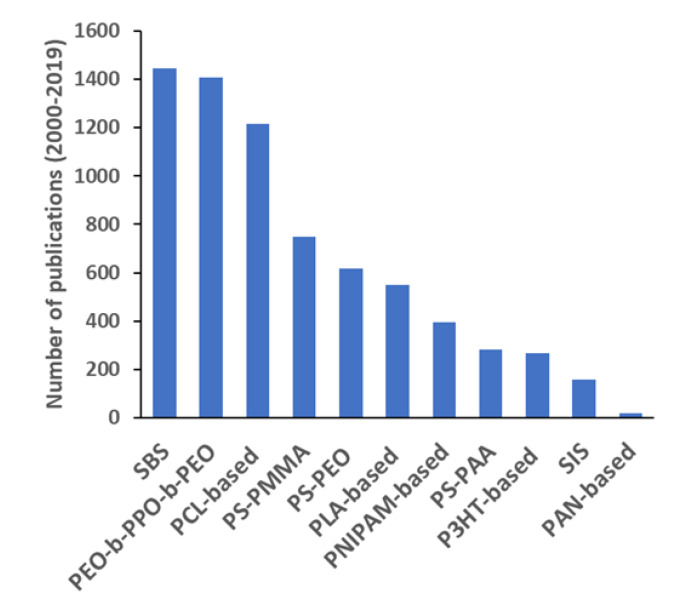
Number of publications related to representative block copolymers from 2000 to 2019.

**Figure 12:**
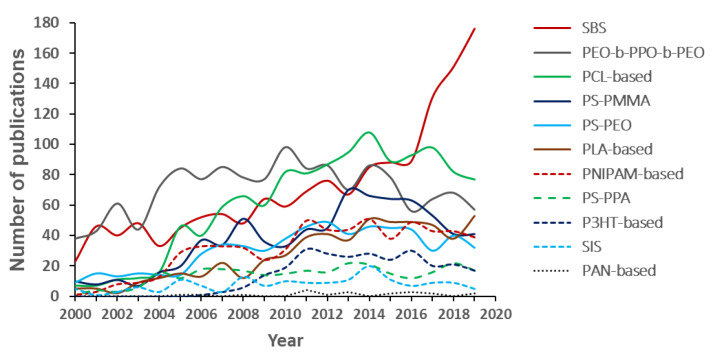
Trend for the number of publications referring to representative block copolymers from 2000 to 2019.

Figure 11 shows the number of publications in the period 2000–2019 by type of block copolymer as directly mentioned in the title of the publication or in the abstract, and considered as representatives of the different classes. The trend of each type over those 20 years is shown in Figure 12. As discussed with respect to Figure 1, this range includes the faster growing period of the field. Although this approach is not exhaustive, it aims to give an idea about the most investigated copolymers and how their interest is evolving throughout the golden age of block copolymers.

Styrene–butadiene–styrene (SBS) block copolymer, one of the few industrially available, appears as the most used over the period, both in investigations covering basic aspects [49] and in industrially oriented publications [50]. As a fact, the total number of publications studying this copolymer is growing steadily, up to a maximum of 176 in 2019. Similar considerations, even though for a smaller number of documents, may be extended to styrene–isoprene–styrene (SIS) block copolymer. The interest for poly(ethylene oxide)–*block*–poly(propylene oxide)–*block*–poly(ethylene oxide) (PEO–*b*–PPO–*b*–PEO) copolymers, and especially for the materials traded as Pluronic©, is mostly related to their biocompatibility and use as vehicles for diagnostic and therapeutic agents [51], and in lesser extent to their application as template, especially for mesoporous silica [15]. From 2002 to 2019, this family of polymers was investigated in 50–100 documents per year. Other highly investigated copolymers, generally composed of biocompatible, biodegradable hydrophobic polymer blocks covalently bonded to a biocompatible hydrophilic block, typically PEO, are those based on polycaprolactone (PCL) [52] and poly(lactic acid) (PLA) [22]. Also these copolymers owe their success to the application as delivery vehicles, confirming once more the central position of this growing sector within block copolymer investigations. During the last 5 years, around 50 articles per year were reporting applications of each of these two classes of amphiphilic copolymers. 

Three other diblock copolymers, such as those based on PS-PMMA [40], PS–PEO, and polystyrene-poly(acrylic acid) (PS–PAA), may be considered as model systems and have long been used for both investigations on basic aspects related with phase separation (in solution or in the solid state, depending on the blocks) or for specific applications, often as a tool for nanomaterial fabrication [53]. In particular, PS–PMMA diblock copolymers appeared in 780 publications over the period (with a maximum of 70 in 2013) and were still the focus of many studies on, e.g., lithographic applications [54]. At the end, three other promising classes of block copolymers are those based on poly(*N*-isopropylacrylamide) (PNIPAM), poly(3-hexylthiophene) (P3HT), and polyacrylonitrile (PAN), especially in publications related to their capacity to respond to temperature stimuli (30–50 publications per year since 2010) [55], in photovoltaic devices (20–30 publications per year since 2010) [56], or as precursor of nanostructured carbons [57], respectively.

## 4. Conclusions and Outlook

Analyzing the bibliometric data on block copolymer research worldwide, from 1952 to 2019, the following conclusions may be drawn:

(1) After the first 20 years with just a few publications per year on block copolymers, and then a slow increase of their number until 1992, the interest grew rapidly in the following decades, reaching a maximum of ca. 2300 publications in 2012. The years between 2013 and 2019, with a constant production of around 2000 documents per year, suggests the achievement of a maturity level (Figure 1).
(2) As common in many fields, original articles were by far the most common type of publications (85.3%) (Figure 2), and English was the predominant language used in 93.9% of documents. Nevertheless, Chinese and Japanese were used in 2.2% and 2.0% of documents, respectively, suggesting the strong interest of China and Japan for block copolymer research.
(3) The top 15 institutions are mainly from the United States, China and Japan; apart from some large national agencies, the Tokyo Institute of Technologies was the most productive institution (Figure 3). Only a small number of companies, mainly from the United States, appear amongst the most relevant institutions. The analysis of publications by country indicates the overall leading position of the United States, accounting for more than one-fourth of documents. After a very long time with the United States dominating the block copolymer research, in the last years, other countries with superior potential became the centers of much greater communities of countries (Figure 7) that show a higher level of international collaboration. In 2019, the Unites States and China were the dual center of the sector, with Japan, South Korea, and Germany occupying the secondary positions.
(4) *Macromolecules* is the journal that published the highest number of block copolymer-related documents and continuously occupied the first position in the last 30 years (Figure 8). Other journals that contributed to the growth of interest for the field are *Polymer*, *Journal of Polymer Science Part A: Polymer Chemistry*, *Macromolecular Chemistry and Physics*, *Macromolecular Rapid Communications,* and *European Polymer Journal*, and, in the recent years, *Polymer Chemistry* or the open access journal *Polymers*, as well as the materials journals *Langmuir* and *Soft Matter*.
(5) The analysis of the top 15 keywords indicated that approximately one-third of publications concerned synthesis (with a special mention for atom transfer radical polymerization) (Figure 9), around 20% mentioned aspects regarding molecular and structural characterization techniques, another 20% explored self-assembly, phase separation, and other morphological aspects, and around 9% investigated hydrophobicity and hydrophilicity. In addition, the keywords ‘micelles’ and ‘nanoparticles’ account for 20.4% of documents; also, the strictly related term ‘polyethylene oxides’, mostly referring to their application in solution, was used in another 20% of publications.
  The changes in the focus of block copolymer research could be envisaged from the evolution of keywords (word clouds in Figure 10). During the first 25 years, investigations mostly concerned essential aspects, and also until the end of the 1990s, the most common keywords were those related to synthesis and basic molecular and structural characterization, being ‘styrene’, ‘butadiene’, and later ‘polystyrenes’ and ‘polymethyl methacrylates’, the most common references to specific block copolymers. In 2009, block copolymers in solution, through the keywords ‘micelles’, ‘drug delivery’, and ‘polyethylene oxides’, focused the attention of the research community, as well as did new types of polymerization techniques. The last analyzed year, i.e., 2019, confirmed the expansion of studies related to drug delivery and, to a minor extent, to a deeper view of self-assembly processes, also through the new developments of specific characterization techniques (X-ray diffraction, electron microscopies, etc.).
(6) The most representative block copolymer types found in publications since 2000 range from the most commonly used SBS to the peculiar block copolymers based on PNIPAM (thermoresponsive systems), P3HT (application in photovoltaics), and PAN (nanostructured carbon precursor), through the commercial Pluronic© family and other amphiphilic copolymers as those based on PCL and PLA (applications as delivery vehicles) (Figure 11). The success of other PS-based copolymers lay in their use as model systems (in particular PS-PAA and PS-PMMA diblock copolymers) or for the fabrication of nanomaterials (again PS-PMMA, and also PS-PEO), the latest application essentially due to their easy self-assembly in thin films to form ordered nanostructures with controlled orientation. With respect to the identification of growing trends, both Pluronic© and especially the industrial SBS, showed a renewed appealing, possibly associated to their easy availability and widespread field of application.

With respect to the initial Feynman-like question on the perspectives for block copolymer research, in our opinion, future trending topics will concern nanomedicine challenges and technology-related applications, with a special role played by the fine-tuning of processing conditions to control the orientation and ordering of mesophase-separated morphologies, to establish complex hierarchical features. A deep look into current investigations makes us speculate on the potential held by some hot papers. In particular, we would like to envisage a bright future for applicative challenges concerning the integration of specific block copolymers into large-scale manufacturing, namely in (i) all-polymer solar cells by fully conjugated donor–acceptor block copolymers [58], (ii) soft-solid electrolytes, as in micellar ion gels for self-healing electrochemical devices [59], (iii) nanofiltration membranes [60], and for the fabrication of (iv) N- and S-doped nanostructured carbons useful in adsorption, catalysis, and energy storage [57] or (v) nanoelectronic devices, e.g., through the optimization of infiltration techniques to be inserted into semiconductor fabrication processing [61]. Moreover, as recently discussed in a more general essay by Binder [62], additional strength to block copolymer synthesis investigations could come from the development of methodology mimicking biological systems, e.g., through the use of enzymes in enzymatic ATRP, or from a deeper tuning of “click” reactions to link large and voluminous molecules. At the end, yes, there is still plenty of room for block copolymer research, especially if the players will be able to strengthen connections, either interdisciplinary and with industry, that enable taking advantage of the huge amount of basic knowledge gathered over almost 70 years of excellent investigations.
